# Using Touchscreen Tablets to Help Young Children Learn to Tell Time

**DOI:** 10.3389/fpsyg.2016.01800

**Published:** 2016-11-17

**Authors:** Fuxing Wang, Heping Xie, Yuxin Wang, Yanbin Hao, Jing An

**Affiliations:** School of Psychology, Central China Normal UniversityWuhan, China

**Keywords:** touchscreen, learning, transfer, children, iPad

## Abstract

Young children are devoting more and more time to playing on handheld touchscreen devices (e.g., iPads). Though thousands of touchscreen apps are claimed to be “educational,” there is a lack of sufficient evidence examining the impact of touchscreens on children’s learning outcomes. In the present study, the two questions we focused on were (a) whether using a touchscreen was helpful in teaching children to tell time, and (b) to what extent young children could transfer what they had learned on the touchscreen to other media. A pre- and post-test design was adopted. After 10 min of exposure to an iPad touchscreen app designed to teach time, three groups of 5- to 6-year-old children (*N* = 65) were, respectively, tested with an iPad touchscreen, a toy clock or a drawing of a clock on paper. The results revealed that post-test scores in the iPad touchscreen test group were significantly higher than those at pre-test, indicating that the touchscreen itself could provide support for young children’s learning. Similarly, regardless of being tested with a toy clock or paper drawing, children’s post-test performance was also better than pre-test, suggesting that children could transfer what they had learned on an iPad touchscreen to other media. However, comparison among groups showed that children tested with the paper drawing underperformed those tested with the other two media. The theoretical and practical implications of the results, as well as limitations of the present study, are discussed.

## Introduction

Touchscreen devices are increasingly prevalent forms of technology used by adolescents and adults. The use of touchscreen technology is also prevalent in early childhood (Cristia and Seidl, 2015). According to a 2013 survey about children’s media use in the U.S., 63% of children from 0 to 8 years old have smartphones to play with and 40% have tablets, most of which use touchscreen technology. The average amount of time children spend using all mobile devices, including those with touchscreens, is 67 min in a typical day. Fifty eight percent of parents have downloaded applications (“apps”) for their children to use on these devices (Common Sense Media, 2013).

There has been an explosion of apps that are claimed to be educational for young children. By 2016, Apple reported that there were over 170,000 apps designed specifically for educational purposes (Apple, 2016). App developers allege that these apps can promote children’s intelligence, help them obtain specific knowledge, and improve their learning performance. However, very few of these so-called “educational” apps have been evaluated and tested (Hirsh-Pasek et al., 2015). Importantly, many of these apps use touchscreen technology, but there are very few studies examining the impact of apps used with touchscreen technology on children’s cognitive and social development (Romeo et al., 2003; Crescenzi et al., 2014; Cristia and Seidl, 2015; Noorhidawati et al., 2015; Huber et al., 2016). In this study, we used a time learning app (Interactive Telling Time, from Apple App Store) to explore how touchscreen influences children’s learning. The topic of reading the time was selected because it was a topic in the Chinese curriculum for kindergarten, and it was determined from the participants’ teachers that children at this grade level had limited knowledge about reading the time.

Retaining new knowledge and skills from interacting with tools and the environment is an important ability for human beings. Compared to traditional media (e.g., printed text), the special feature of touchscreen technology is finger-based touch or interactivity. Christakis (2014) summarized these qualities by saying that touchscreen devices are interactive, tailorable, and progressive compared to traditional toys. Hirsh-Pasek et al. (2015) suggested that touchscreen apps should be designed to promote active, engaged, meaningful, and social interactive learning. Studies have shown that the embodied touching and interactivity have significant effects on learning (Agostinho et al., 2015; Dubé and McEwen, 2015; Moser et al., 2015; Wang et al., 2015; Huber et al., 2016). The embodied cognition theory proposed that cognitive processes are rooted in the body’s interactions with the world, and cognition should be understood in the context of its relationship to a physical body that interacts with the world (Wilson, 2002; Shapiro, 2010). For example, one study showed that explicit instructions to trace out elements of geometry worked examples with the index finger could enhance learning outcomes (Hu et al., 2015). Recently studies explored the relation between physical interactions with a touchscreen device and learning improvement. Dubé and McEwen (2015) asked participants to complete a number line estimation task by either tapping or dragging on a tablet. Results indicated that participants in the drag condition were more accurate than those in the tap condition. Similarly, a study with worked examples on mathematical problem-solving found that finger tracing as physical movement and interaction with the environment could enhance leaning performance (Agostinho et al., 2015). The first goal of the present study was to determine whether 5- and 6-year-olds showed better ability to tell time after using a touchscreen app designed to teach clock reading.

Many researchers have explored the possibility that the touchscreen promotes learning (Romeo et al., 2003; Crescenzi et al., 2014; Wong, 2015). One study with adults found that the interactive feature (e.g., dragging an object across the screen) could improve mathematical learning performance (Dubé and McEwen, 2015). Wang et al. (2015) showed that iPad apps can not only improve students’ learning performance, but also increase motivation for language learning. Studies with 8- to 11-year-olds showed that children who learned about temperature graphs by tracing their finger on the iPad touchscreen showed better performance than a non-tracing (viewing) group (Agostinho et al., 2015). Moreover, researchers have argued that touchscreen tablets such as the iPad have the potential to promote children’s literacy, such as alphabet knowledge, print concepts, and emergent writing (Neumann and Neumann, 2014). Berkowitz et al. (2015) found that using educational apps at home improves children’s math achievement at school. In short, all these studies indicate that the touchscreen has positive effects on learning. For the present study, all the children learned how to tell time on an iPad with an interactive app, but were tested with three different media: iPad, toy clock, and paper.

However, the educational effect of touchscreen technology has also been questioned in some studies. For example, Dundar and Akcayir (2012) did not find differences in 11- to 12-year-olds’ reading speed or reading performance via learning with printed books compared to touchscreen tablets. Chen et al. (2014) found that college students’ reading performance was similar for both touchscreen tablet and paper. An investigation suggested that individuals who think more intuitively and less analytically when given reasoning problems are more likely to rely on internet through their Smartphones (Barr et al., 2015). As a consequence, it is possible that not all touchscreen technology has positive effects on cognition, with benefits depending on what we use and how we use it.

It should be noted that previous studies tried to compare touchscreen with other media (e.g., paper, computer) and other learning methods (e.g., traditional semantic-map method) to find which one is more effective (Dundar and Akcayir, 2012; Chen et al., 2014; Wang et al., 2015). By comparing the effects of touchscreen and other media, there could be no direct indication of whether the touchscreen itself has a positive effect. Therefore, the present study used a pre-test and post-test design to directly investigate whether touchscreen can improve learning performance, using the specific task of learning to tell time. The advantage of pre- and post-test is that researchers can determine the effect of an experimental intervention by post-test score minus pre-test score. In this study, we used a pre- and post-test design to explore whether children’s performance can be improved after they use an iPad touchscreen app to learn how to tell time on a clock.

An important goal of touchscreen learning is that children be able to transfer the knowledge they learned from interaction with the touchscreen and use it to solve problems in real life. Moser et al. (2015) found that 2.5- and 3-year-old children had transfer deficits on a puzzle assembling task, in that they could not transfer very well from touchscreen to a real 3D situation. However, Huber et al. (2016) found that 4- to 6-year-olds could transfer what they learned about solving a problem (Tower of Hanoi) on touchscreen to physical objects. In summary, the older children (more than age 4) have acquired the ability to transfer from touchscreen media to a situation not involving the touchscreen. Based on this literature, the second goal of this study was to test the extent to which the test medium affects transfer of learning. We tested transfer of learning to a toy clock (which is similar to the iPad clock and to real life clocks) and to a drawing of a clock on paper (with paper being the most common medium used in classrooms).

In this study, we chose the app “Interactive Telling Time” as an iPad touchscreen learning material and tested 5- to 6-year-old children’s transfer of learning from iPad to different media. A pre- and post-test design was used in which all children learned about telling time by using the touchscreen, and then were tested using one of three methods. Based on the interactive feature of the iPad and the app we used (Dubé and McEwen, 2015; Hirsh-Pasek et al., 2015), we predicted that learning with the iPad touchscreen would be helpful, with post-test scores being higher than pre-test. Moreover, based on similarities and differences among the original touchscreen learning device and the test materials, we predicted that testing on the iPad touchscreen would produce better performance compared to the toy clock and paper, and the toy clock would be better than paper.

## Materials and Methods

### Participants and Design

A total of 65 (32 girls) 5- to 6-year-old children (*M* = 70.4 months, *SD* = 4.0) without history of neurological or psychiatric illness participated in the current study. They were recruited from a preschool in Wuhan, China. All children used an iPad touchscreen to learn to read a clock and then each participant was assigned to one of three post-test assessment groups: iPad touchscreen (*n* = 22, *M*_age_ = 71.3 months, *SD* = 3.5, 9 girls), toy clock (*n* = 21, *M*_age_ = 70.8 months, *SD* = 4.5, 12 girls), or paper drawing (*n* = 22, *M*_age_ = 69.3 months, *SD* = 4.0, 11 girls). No difference was found among groups on age [*F*(2, 62) = 1.48, *p* > 0.05]. All children were from Chinese middle-class families (participants’ family income was the equivalent of 20,000 to 40,000 USD per year) and they were given stickers for their participation. All parents and teachers signed informed consent forms. This study was approved by the Institutional Review Board of Central China Normal University.

### Materials

**FIGURE 1:**
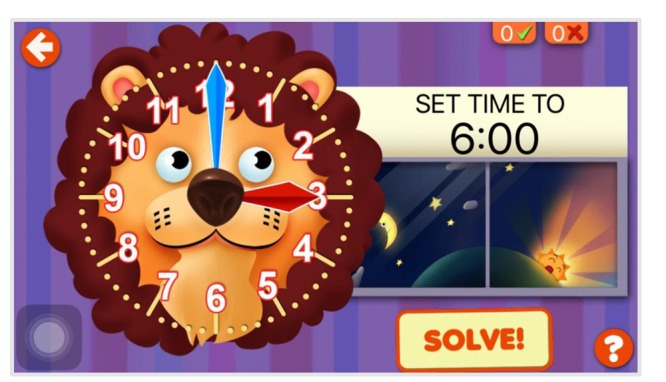
**Snapshot taken from Interactive Telling Time “SET the Time” on the iPad**.

Each participant learned to read the time on an iPad Air 2 touchscreen using an app “Interactive Telling Time.” Considering the complexity of children’s time conceptions and the potential difficulty of teaching them to tell time (Burny et al., 2009, 2011, 2013; Labrell et al., 2016), only the hour times (e.g., 1:00, 9:00, 12:00, which we defined having minute hand on 12) and half-hour times (e.g., 1:30, 3:30, 6:30, which we defined having minute hand on 6) were presented in the format of a 12-h clock to reduce the difficulty of the learning material. Ante meridiem (a.m.) and post-meridiem (p.m.) were not differentiated. The learning material ran on an iPad app named “Interactive Telling Time” (GiggleUp Kids Apps and Educational Games Pty Ltd). This app provided multiple modules, including several learning modules and test modules. One of the learning modules, “SET the Time,” was selected to present the material (see **Figure 1**). Details of this module were as follows.

At the right center of the interface, there was a target time region that had a white background. Trials of the target time were presented in this area in visual text form [e.g., “SET TIME TO 6:00” (“[scale=.7]./fig/img001.eps 6:00” in Chinese)], accompanied by narration in a female voice when a learning trial initially appeared. If a participant forgot what the current target time was during the trial, he/she could touch the white region for a second narration.

The left side of the interface showed a colored lion clock. The clock face had 12 numbers, a small red hour hand, and a big blue minute hand. No second hand was included. Before the initial touch of each trial, the time on the clock face was a random “wrong” hour time or half-hour time that was inconsistent with the target time (e.g., 5:00). Learners were required to adjust the “wrong” time on the clock face to match the target time through touching and rotating the clock hands. Any adjustment of the small hand or big hand would activate a time-telling voice from the app (e.g., “five past six!”).

A “SOLVE!” button was located at the bottom right corner of the interface. Once participants thought they had adjusted the small hand and big hand to the right locations, they could touch the button. If the adjusted time was correct (i.e., consistent with the target time), spoken feedback was provided in a cheerful voice (e.g., “Well done!”), then the app advanced to the next learning trial. If the adjusted time was wrong, a warning tone would be given and the present trial would not disappear, reminding the participants that they had not adjusted the time correctly and further adjustments were needed until the target time was set.

### Apparatus

Three kinds of apparatuses were used to test children’s learning outcomes.

#### iPad Touchscreen Test Apparatus

**FIGURE 2:**
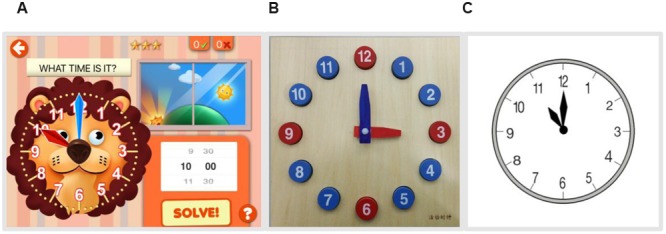
**The apparatuses were used in the post-test of three different groups. (A)** Test module “What’s the time?” on the iPad touchscreen app used in the iPad test group. **(B)** A real toy clock used in the toy clock test group. **(C)** One of the test trials used in the paper test group.

For the iPad test group, the apparatus and app were the same as the ones used in the learning phase, except that, we switched to the test module “What’s the time?” (see **Figure 2A**). The clock on the touchscreen app had a lion face at the center. Again, the left side of this test interface showed a clock face identical to the learning module. No second hand or other markers for seconds (e.g., graduated bars for second hand) were included.

#### Toy Clock Test Apparatus

For the toy clock test group, a real colored wooden toy clock was used, with a size of approximately 25 cm × 25 cm × 5 cm (width × height × depth; see **Figure 2B**). Unlike the clock on the touchscreen app, which had a lion face at the center, the clock face on the toy was plain. It had 12 numbers, a small red hour hand, and a big blue minute hand. No second hand or other markers for seconds were included.

#### Paper Test Apparatus

For the paper test group, the clock face with 12 numbers of each test trial was printed in black and white on A4 paper, just like what we saw in the real classroom test (see **Figure 2C**). Similarly, the design of the clock face was simple. No second hand or other markers for seconds were included.

### Procedure

The present study consisted of five consecutive phases: pre-test, instruction, learning, interference, and post-test. The whole procedure lasted approximately 20 min.

#### Pre-test Phase

First, the experimenter asked children to orally report the 12 numbers that were arranged in a pseudo-random order on the paper (i.e., 1, 8, 3, 10, 2, 11, 5, 6, 7, 12, 4, 9). Second, one clock face printed in black and white was presented to check whether they could read the time. Then, the children were asked about their touchscreen experience (e.g., “How often do you play on an iPad, smartphone, etc.?”) using a four point Likert scale. Zero points were received if the answer was “Never,” and three points if “Every day.” Thereafter, we presented children with 12 clock faces (similar to **Figure 2C**) with different times on a printed paper. Six of them were hour times, and six were half-hour times. Children were asked what time it was on each clock face one by one. One point was awarded for each correct answer, yielding a maximum of 12 points. Children whose pre-test scores were no more than eight were asked to attend this research.

#### Instruction Phase

A clock face on the iPad touchscreen app was shown to make sure that the children could correctly distinguish between the small hand and the big hand (e.g., Look, there are two hands on this clock face, right? Would you mind pointing out which one is the small hand and which is the big one?). To make participants familiar with the position and arrangement of each number on the clock face, the experimenter read out those 12 numbers in a clockwise direction and asked them to point out the numbers 12 and 6. Then, a simple instruction was given to familiarize the children with the hour times. Specifically, a rule to recognize hour times (i.e., When the big hand is pointing straight up at the number 12, we say the word “o’clock!”) and two examples (e.g., You see, the big hand is pointing straight up at the number 12 and the small hand is pointing at 9, then we say “9 o’clock”) were given to the children. Next, a similar instruction was given for the familiarity of half-hour times.

#### Learning Phase

Children spent 10 min alone learning to read the time on the iPad touchscreen app (Module: “SET the Time,” see **Figure 1**). As for the 10 min learning time, first, we consulted teachers in the kindergarten and found that duration of studying the knowledge of clock in the classroom is about 10 min; second, we ran a pilot study with four children before we conducted this study, and found that there was a limited time period during which children could concentrate on what they were studying. Therefore, we finally set 10 min as the learning time. The number of learning trials was unpredictable. The experimenter recorded the number of trials of the learning phase.

#### Interference Phase

After the learning phase, 3 min were given to the children to write down their names by themselves and to have a rest. Based on the pilot study, we found two of the children would mutter or repeat what they had learned after learning. Thus, we add an interference phase to control the short-term memory influences.

#### Post-test Phase

Twelve clock faces with different time points were successively shown to the participants in a random order. Half of them were hour times and half were half-hour times. Participants were required to orally report the time as loudly as possible. Children were tested using one of three kinds of media. The iPad test group was tested on the iPad touchscreen app (Module: “What’s the time?,” see **Figure 2A**), but the children were not allowed to touch the screen in the post-test phase. Every time a test trial appeared, participants were asked “What time is it?” by the app system. The toy clock and paper test groups were tested on a real toy clock (see **Figure 2B**) or the paper (see **Figure 2C**), respectively. The same question was asked by the experimenter.

## Results

All 65 participants knew the 12 numbers and the clock face. Bonferroni adjustments were made when conducting *post hoc* multiple comparisons. Effect sizes were reported as partial η^2^ values (η_p_^2^). One-way ANOVAs revealed no difference across test media groups in prior touchscreen experience [*F*(2, 62) = 0.27, *p* > 0.05], but a significant difference on number of learning trials [*F*(2, 62) = 3.78, *p* < 0.05]. *Post hoc* multiple comparisons showed that children in the paper test group had more learning trials than children in the iPad test group. There was no significant difference between the toy clock and paper test groups, as well as iPad and toy clock test groups. Descriptive values are shown in **Table 1**. Following are the results for three dependent variables: (a) score for telling time; (b) acquisition size; (c) acquisition efficiency.

**Table 1:** Means and standard deviations as a function of test media.

Variables	Test media (*SD*)		
	iPad	Toy clock	Paper
Touchscreen experience (0–3)	1.18 (1.01)	1.38 (0.74)	1.32 (0.95)
Pre-test score (0–12)	2.68 (2.82)	3.19 (2.93)	3.23 (2.41)
Number of learning trials	9.73 (6.71)	14.76 (9.23)	16.14 (8.31)
Post-test score (0–12)	8.32 (3.33)	8.38 (3.20)	6.23 (3.64)
Acquisition size (AS)	5.64 (3.19)	5.19 (3.49)	3.00 (2.69)
Acquisition efficiency (AE)	0.95 (1.27)	0.45 (0.42)	0.20 (0.18)

**FIGURE 3:**
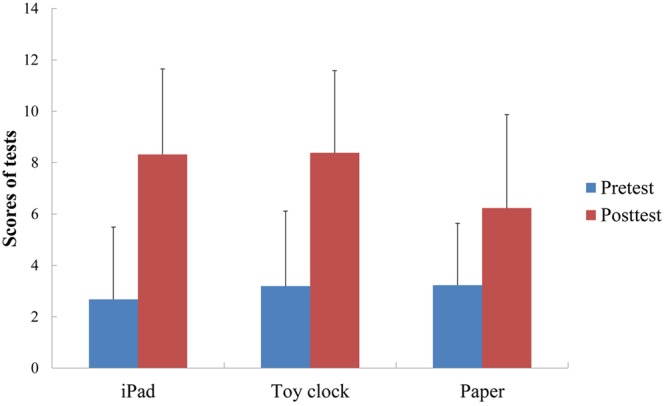
**Means of pre-test and post-test scores (with *SD*) as a function of test medium**.

A repeated measures ANOVA with test medium (iPad touchscreen, toy clock, and paper) as the between-participants variable, test session (pre-test and post-test) as the within-participants variable, and number of learning trials as the covariate was conducted on test scores (see **Table 1** and **Figure 3**). Score on the clock-reading was set as the dependent variables. The results showed a main effect of test medium [*F*(2, 61) = 3.97, *p* < 0.05, η_p_^2^ = 0.12], and a main effect of test session [*F*(1, 61) = 12.71, *p* < 0.001, η_p_^2^ = 0.17]. However, these effects had to be interpreted in terms of the significant interaction between test medium and test session [*F*(2, 61) = 8.13, *p* < 0.001, η_p_^2^ = 0.21]. Analysis of the simple effects of test session for each test medium type indicated that children in all groups had higher post-test scores than pre-test scores [iPad test group: *F*(1, 62) = 71.23, *p* < 0.001; toy clock test group: *F*(1, 62) = 57.66, *p* < 0.001; paper test group: *F*(1, 62) = 20.18, *p* < 0.001]. In addition, analysis of the simple effects of test medium type for each test session revealed no significant difference among the three groups on pre-test scores [*F*(2, 62) = 0.27, *p* > 0.05]. There was a marginally significant difference for post-test [*F*(2, 62) = 2.84, *p* = 0.066]. The paper group was significantly worse than the iPad group and toy clock group according to Newman–Keuls *post hoc* test (*p*_s_ < 0.05).

Acquisition size (AS) was calculated by subtracting pre-test scores from post-test scores (see **Table 1**). Taking AS as the dependent variable, a one-way ANOVA revealed a significant difference among groups [*F*(2, 62) = 4.45, *p* < 0.05, η_p_^2^ = 0.13]. *Post hoc* multiple comparisons indicated that children in the iPad and toy clock test groups outperformed those in the paper test group (iPad vs. paper: *Mean Difference* = 2.64, *p* = 0.021; toy clock vs. paper: *Mean Difference* = 2.19, *p* = 0.076). However, the difference between the toy clock and paper was marginal. No difference was observed between the iPad and toy clock groups (*Mean Difference* = 0.45, *p* > 0.05).

Further, acquisition efficiency (AE) was calculated by dividing AS by the number of learning trials (see **Table 1**). Taking AE as the dependent variable, a one-way ANOVA revealed a significant difference among groups [*F*(2, 62) = 5.26, *p* < 0.01, η_p_^2^ = 0.15]. *Post hoc* multiple comparisons showed that children in the iPad touchscreen test group outperformed those in the paper test group (*Mean Difference* = 0.75, *p* = 0.007). No difference was observed between the iPad touchscreen and toy clock groups (*Mean Difference* = 0.50, *p* > 0.05) or the toy clock and paper groups (*Mean Difference* = 0.25, *p* > 0.05).

## Discussion

Although touchscreen devices are prevalent in children’s lives and influence children’s development (Cristia and Seidl, 2015; Bedford et al., 2016), there are few studies examining the effects of touchscreen on children’s cognition and learning. In the present study, we used a pre- and post-test paradigm to examine whether using a touchscreen iPad could facilitate young children’s learning to tell time, and whether they could transfer this learning from iPad to different media (i.e., a physical object and paper). The results showed that the post-test score was higher than pre-test after children used an iPad touchscreen app to learn how to read time on a clock. This result is consistent with our hypothesis, indicating that 5- to 6-year-old children could benefit from touchscreen technology to learn this skill. Additionally, we found that 5- to 6-year-old children’s new knowledge about telling time transferred very well from iPad to iPad and from iPad to the physical toy clock. The findings suggest that touchscreen devices or interactive touchscreen educational apps not only facilitate young children’s acquisition of knowledge and skills, but also can promote transfer of new knowledge to solve problems using different media. This study moves the research from a general focus on apps to a focus on one app in particular. Implications of this study are useful for parents and teachers, who could use touchscreen technology to encourage children’s active learning.

Compared with printed books and video, one special feature of touchscreen is interactivity. Children could tap, drag, and touch the objects on the touchscreen and get a response from the objects. From the view of embodied cognition, cognitive processes are deeply rooted in the body’s interactions with the world (Wilson, 2002; Shapiro, 2010). Embodied cognition provides a good framework to explain why touchscreen facilitates young children’s learning. A touchscreen, such as an iPad, gives children opportunities to interact with what they are learning about, not just watch and listen. Children’s engagement with touchscreen apps provides motor, visual, and acoustic information, and benefits memorization (Agostinho et al., 2015; Noorhidawati et al., 2015). In this study, children could move their finger to drag the clock’s minute hand and hour hand to set the time. If they did not get the right answer, they would get a voice reply telling them to try again. These exchanges with the touchscreen device are thought to be the process that promotes children’s learning.

The post-test scores indicated that children could easily transfer what they learned from the iPad touchscreen to the toy clock and paper. These results were consistent with the hypothesis. Huber et al. (2016) found that 4- to 6-year-old children could learn how to solve Tower of Hanoi on an iPad touchscreen and subsequently apply this learning to physical objects. When children actively engaged in the touchscreen learning process, learning was enhanced (Hirsh-Pasek et al., 2015). Unlike passive learning from video, the touchscreen used in this study was interactive and informative, and children were willing to engage in learning.

However, after learning with the iPad touchscreen, children in the toy clock assessment group performed as well as those assessed using the iPad. This is inconsistent with our hypothesis. The result is also inconsistent with a previous study, which found that 3-year-olds showed lower transfer from touchscreen to physical objects (Moser et al., 2015). The researchers argued that young children could encode the information from the touchscreen but could not retrieve the information on new media or environments because they lacked memory flexibility. However, the memory flexibility and the cognition of children more than 3 years old have reached a new level (Zelazo et al., 1999; Dickerson et al., 2013). In this research, we recruited 5- to 6-year-old children. They could transfer knowledge very well between different media.

Part of the reason for the transfer seen in this study is that the real toy clock was similar to the clock on the iPad app in shape and color. These similarities could benefit the learning and transfer. As for the group tested with a paper drawing, the improvement of learning was the lowest. Analysis of the simple effects of test medium type for each test session revealed that children in the iPad and toy clock test groups outperformed those in the paper test group. In addition, results from AS and AE also showed that children assessed using a paper drawing acquired the least and had the lowest efficiency. The reason might be that the post-test material on paper was printed in white and black, had a very simple shape, and was far from the learning material on the iPad touchscreen app. Therefore, these features of the paper material may hinder children’s transfer. For example, studies with multimedia learning showed that the shape, color and anthropomorphism of material could affect learning performance (Um et al., 2012; Plass et al., 2014; Park et al., 2015). Bright colors and anthropomorphic shape in the iPad group and the toy clock group could facilitate learning performance. This speculation still needs to be further verified.

Several limitations of this study should be acknowledged. First, it will be important in future research to ask children to report which type of medium they liked. This will give more information to explain how the assessment format might influence transfer of learning from the touchscreen to other media. Second, all the materials should be matched with regard to color, shape and anthropomorphism, to provide a more valid test of the effects of the touchscreen *per se*. Third, our learning task was telling time, and future research should evaluate the extent to which other skills learned on touchscreen can be applied to different media. Besides telling time, a variety of apps should be examined to generalize the conclusions about the promotion of touchscreen on learning. Finally, other media types (e.g., video, TV) are still to be tested. This limited intervention showed positive outcomes. It is unclear whether more extensive use could lead to negative effects, a question that still needs empirical study.

## Author Contributions

FW developed the study concept. All authors contributed to the study design. FW, HX, YW, JA, and YH performed the experiment, and HX conducted the statistical analyses and interpreted the results. FW, HX, and YW were primarily responsible for writing the manuscript, with all remaining authors providing critical revisions. All authors approved the final version of the manuscript for submission.

## Conflict of Interest Statement

The authors declare that the research was conducted in the absence of any commercial or financial relationships that could be construed as a potential conflict of interest.
